# Simple, rapid, and efficient purification of M13 phages: The Faj-elek method

**DOI:** 10.1371/journal.pone.0325621

**Published:** 2025-06-06

**Authors:** Gizem Kılıç, Esma Aybakan, Neda Tatlıoğlu, Gökçen Özgün, Tanıl Kocagöz, Erkan Mozioğlu

**Affiliations:** 1 Department of Medical Biotechnology, Institute of Health Sciences, Acibadem Mehmet Ali Aydinlar University, İstanbul, Türkiye; 2 Department of Medical Microbiology, Faculty of Medicine, Acibadem Mehmet Ali Aydinlar University, İstanbul, Türkiye; Council for Scientific and Industrial Research, SOUTH AFRICA

## Abstract

M13 bacteriophages (phages) are used as very important tools in molecular biology, biotechnology and, nanotechnology. Many methods have been developed so far for the purification of these phages. However, it is important that phages retain their infecting properties, especially in biotechnological applications such as phage display technology. The most widely used is the PEG precipitation method, but it has some limitations such as impurities and reduced infectivity. To overcome them, we developed a new method for purification of M13 bacteriophages using syringe filters made of cellulose acetate membranes with a pore diameter of 0.22 µm. Phages were aggregated so that they could remain on the filters and for this purpose, the pH of the phage cultures was reduced to 3. The phage cultures were filtered and then the phages were recovered in tris-buffered saline (TBS) buffer (pH 10.5) by reversing the filter. The recovery rate was 250% higher than the standard PEG method. This new Faj-elek method offers an alternative to existing methods, allowing cheaper, easier and faster purification of M13 phages using syringe filters available in every research laboratory.

## Introduction

Bacteriophages (phages) are viruses infecting bacteria and are estimated to number 10^31^, which makes them the most numerous entities in the globe [1]. Phages are divided into 3 morphological classes: Filamentous, icosahedral and enveloped phages [2]. Filamentous phages have a parasitic life, meaning that they enter bacteria and propagate themselves without lysis [2]. These phages can infect various Gram (-) bacteria such as *Escherichia coli*, *Salmonella*, *Pseudomonas*, *Vibrio thermus*, and *Xanthomonas* [2]. M13 phages, a member of the filamentous phages, are widely used in biotechnology applications due to their single-stranded circular DNA molecules and relatively simple capsids [2]. M13 phages are 6 nm wide and 900 nm long [3]. When subjected to an external induction, they spontaneously assemble to form long rod-shaped and monodisperse structures in response, allowing them to form unique hierarchical nanostructures [3]. These unique morphological features of M13 phages offer great advantages for their use in nanotechnology. Phage display technology, developed by George Smith in 1985 and awarded the Nobel Prize in Chemistry in 2018, has become one of the most widely practiced applications for the selection of target-specific peptide aptamers based on the use of M13 phages [4]. Beyond their use as antimicrobial agents, M13 phages have been used as nanocarriers in drug delivery, tissue regeneration such as bone and skin, biosensing, because their surface proteins can be easily modified by changes in the genome, thus enabling the display of multiple peptides [5,6].

These wide applications of M13 phages in nanotechnology and biotechnology make it important to obtain pure M13 phages. The production of phages can quite straightforward. Due to the lack of lytic properties, a large number of phages are released into the culture medium within a few hours in the infected *E.coli* bacterial culture. By centrifugation, phages can be separated by precipitating the bacteria. In order to purify these phages in the culture medium by removing the waste of the bacterial culture, different methods have been used as follows.

In the production of M13 phages in large quantities on a research or industrial scale in laboratories, bacteria are used as factories and phages are obtained by purification from these phage cultures. There have been many different methods developed for the purification or concentration of phages and listed in the review article by Fouladvand et. al., (2020). Density-based methods including ultracentrifugation, polyethylene glycol (PEG), spermidine or isoelectric-based precipitation methods as well as chromatographic methods are available [7]. However, all these applications have their advantages as well as some disadvantages such as using of complicated and expensive devices. Therefore, PEG precipitation is one of the most widely used methods in most research laboratories [8,9]. This method is not very complex but time consuming when considering applications with different holding times (in a cold room for 4–5 hours or overnight) [8–10]. Another important problem is that the phages obtained are co-purified with PEGs and if necessary, they need to be re-purified by additional methods such as CsCl density gradient centrifugation [11]. As another approach, precipitation of neutralized phages at the isolectric point is an option, but There are some concerns due to contamination of the co-precipitated molecules with other molecules [7,12–14].

Unlike all of these, there are remarkable studies in the scientific literature on the use of filters composed of charged membranes for the retention of viruses in samples taken from air, groundwater, seawater, lake waters, rivers and sewage waters for the determination and prevention of public health risks, as well as in the preparation of medical products such as vaccines [15–18].

In this regard, we investigated and optimized the use of 0.22 μm injector filters for the purification of M13 phages, which are of great importance in molecular biology and biotechnology since a syringe and syringe filter are readily available in almost every research laboratory. This innovative approach offers a rapid and simple method for the purification of M13 phages with very high recovery and purity. Therefore, it will be a great convenience for all researchers in this field and could replace other existing methods that are widely used. We called this method the Faj-elek Method meaning phage sieve.

## Materials and methods

### Materials

*E. coli* ER2738 and M13 phage (M13KE, a derivative of M13mp19) were obtained from NEB, USA. isopropyl β-d-1-thiogalactopyranoside (IPTG) and 5-bromo-4-chloro-3-indolyl-β-D-galactopyranoside (X-gal) were bought from Goldbio. Other chemicals such as Luria Bertani (LB) medium, PEG, etc. were bought from Sigma, USA. Scanning electron microscopy (SEM with Quatro ESEM) was from Thermo Fisher Scientific, USA and a vacuum sputter coater (EM ACE200) was from Leica, Germany. Syringe filters (Minisart, 16534-K) were obtained from Sartorius, Germany.

### Optimization of Faj-elek method and proof of concept

#### Phage amplification.

This study was done by modifying previous studies in the literature [19]. Briefly, *E. coli* ER2738 strain was grown as starter culture in an LB medium containing 20 µg/mL of tetracycline, at 37°C at 200 rpm by shaking overnight. The next day, it was transferred to a large volume of fresh culture medium (by 1:100 dilution) and infected with M13 phages. The phage culture was incubated at 37°C at 250 rpm for 5 hours by shaking. Then, bacterial cells were removed by centrifugation and the supernatant was transferred into a fresh tube. The supernatant was incubated at 60° C for 1 hour to inactivate the remaining bacterial cells. The death of the bacteria was confirmed by inoculation on sterile medium.

#### Phage counting: Top Agar method.

Phage counting was performed by top agar method [19]. Briefly, *E.coli* ER2738 bacteria were grown to the mid-log phase (OD600 ~ 0.6) at 37°C at 200 rpm by shaking in an LB medium containing 20 µg/mL tetracycline. Then, 200 µL of the bacteria were infected with the 10 µL of the phage sample and incubated for 5 minutes at room temperature. The infected cells were transferred to tubes containing 3 mL of molten top agar. Cultures were mixed and poured onto pre-warmed LB agar media including 1 mM IPTG, 0.2 mg/mL X-gal, and 20 µg/mL tetracycline in final concentrations. Plates were incubated overnight at 37°C. Blue plaques indicating phage-infected bacteria were enumerated as phage forming units (PFU).

#### Filtration of phages: The Faj-elek method.

M13 phages, with a width of 6 nm and a length of 900 nm, are very thin and easily filtered through 0.22 µm or 0.45 µm pore diameter filters and pass into the lysate [3,14,20]. Therefore, phage cultures can be filtered through these filters to get rid of bacteria in biotechnology applications [20,21]. In addition, M13 phages aggregate at low pHs and form nanostructures of larger sizes [22] So, in order to determine the pH value allowing the phages to remain on the 0.22 µm pore diameter filter in this method, the phage culture was adjusted at different pH values between 7–3 by adding HCl and then was diluted to 10^−5^ in LB media at the same pH values. The pH adjustment was performed using pH papers to avoid contamination of the phage culture with the pH probe. For this purpose, samples of the culture for pH measurement were taken in a separate tube, measured with pH paper and this portion was discarded after the measurement, so that the culture was not contaminated in any way. The phage concentration in the primary culture used for the different pH experiments averaged 3.5x10^8^ phage/mL, before dilution.

**Fig 1:**
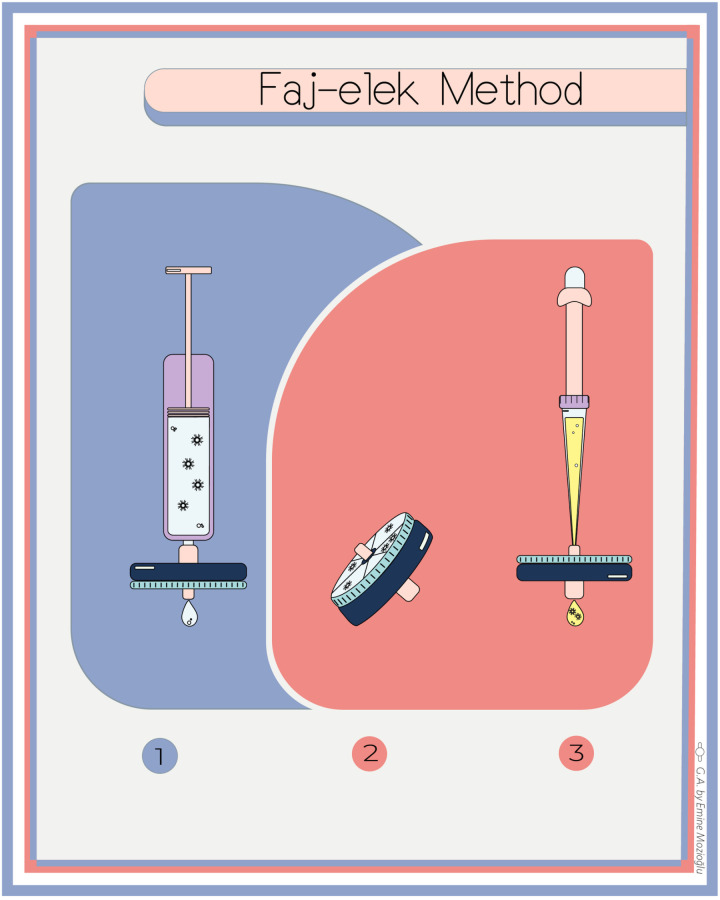
Graphical abstract of the Faj-elek method. 1) Filtration of phage culture **in the forward direction**; 2) Inverting the filter; 3) Filtration of phage culture **in the reverse direction**. The graphic was designed by Emine Mozioğlu.

In order to condition the filters (Minisart, 16534-K, Sartorius), 5 mL each of LB medium at the pH desired for the study was passed through the filters before filtering the phages. After 5 mL each of pH adjusted phage culture was filtered, the filter was inverted and phages were eluted by passing 5 mL of 50 mM glycine solution (pH 10.5) buffer through the filter in the opposite direction. Consequently, the phages passing down the filter and the phages remaining on the filter were cultured with the top agar method and the blue phage plaques were enumerated for comparison. The pH experiments were repeated with three independent filters and the results were calculated as averages. The Faj-elek Method was performed in three steps as illustrated by the graphical abstract (Fig 1):

### Comparison of methods PEG and Faj-elek

Phage amplification was performed as described above. Then, the phage culture (8 mL) was subjected to both the PEG Method and the Faj-elek Method as described below.

#### PEG method.

Four volumes of phage culture were mixed with one volume of PEG solution (2.5 M NaCl and 20% PEG-8000 (w/v)) [23]. After incubation at 4° C for 1 hour, Phages were precipitated by centrifugation at 12,000xg for 15 minutes at 4°C and the supernatant was removed. The pellet was resuspended with TBS buffer (20 mM Tris, 150 mM NaCl, pH 7.6) in 1:16 ratio of the initial phage culture and the supernatant was transferred to clean tubes after centrifugation. The PEG solution was added in the ratio 1:5. After incubation on ice for 15 minutes and then centrifugation at 12,000xg for 10 minutes, the supernatant was removed. The pellet was resuspended with 100 µL of the TBS buffer. Accordingly, phages precipitated with PEG were cultured with top agar method and blue phage plaques were enumerated for comparison. The PEG experiments were performed with three independent replicates and the results were calculated as averages.

#### The Faj-Elek method.

1.5 M NaCl was added to the phage culture and incubated for 15 minutes at room temperature. Then pH of the phage culture was adjusted to 3 by adding HCl acid and they were incubated at room temperature for 15 minutes. In order to condition the filters (Minisart, 16534-K, Sartorius), 5 mL each of LB medium including 1.5 M NaCl at the pH 3 was passed through the filters before filtering the phages. After 8 mL each of pH adjusted phage culture was filtered, the filter was inverted and phages were eluted by passing 8 mL of 50 mM glycine solution (pH 10.5) buffer through the filter in the opposite direction. Accordingly, the phages remaining on the filter were cultured with the top agar method and the blue phage plaques were enumerated for comparison. The pH experiments were repeated with three independent filters and the results were calculated as averages.

### SEM and elemental analysis

Scanning electron microscopy (SEM) was performed with Quatro ESEM (Thermo Fisher Scientific) and elemental compositions were determined with Energy Dispersive X-ray Spectroscopy (EDS). EDS works by sending an electron beam onto the sample, which causes the sample to emit X-rays. Elements emit X-rays at different energies, therefore it is possible to find out which elements are present when measuring them. EDS provides a rapid identification of the elements on the surface of a material when using SEM. For SEM images, samples were sputter-coated with 40 nm thick gold via a vacuum sputter coater (Leica EM ACE200).

## Results

### Optimization of Faj-elek method and proof of concept

**Fig 2:**
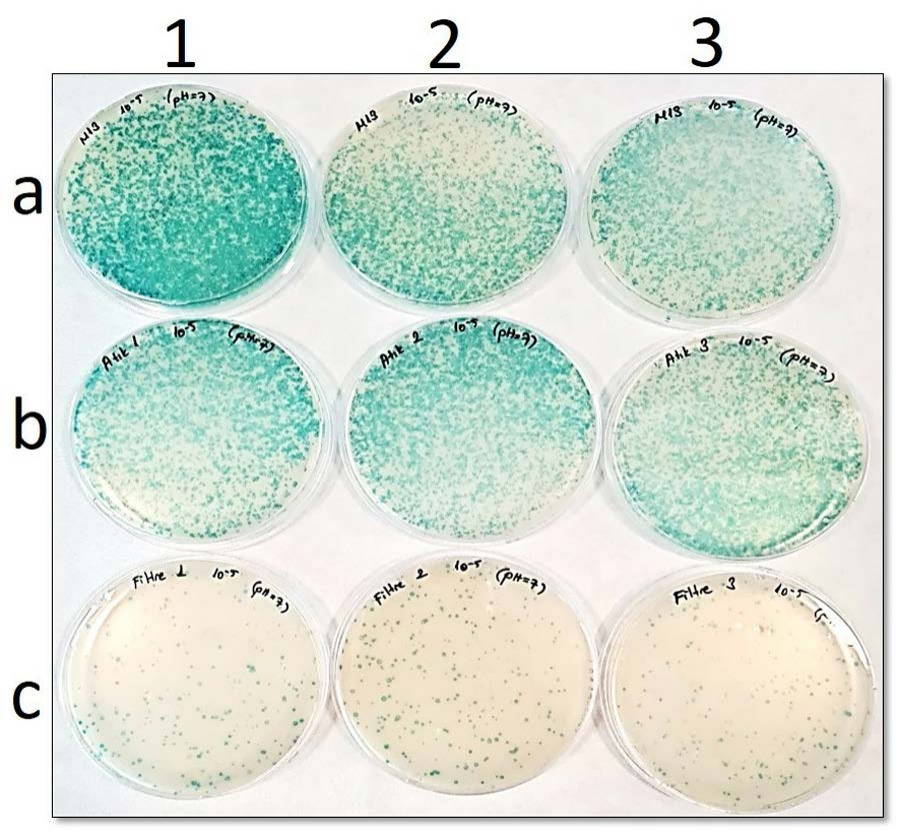
Comparison of phage numbers by the Faj-elek method at pH 7. a) Before filter treatment; b) Phages passing to the lysate; c) Phages remaining on the filter. 1,2, and 3 shows the number of repetitions.

**Fig 3:**
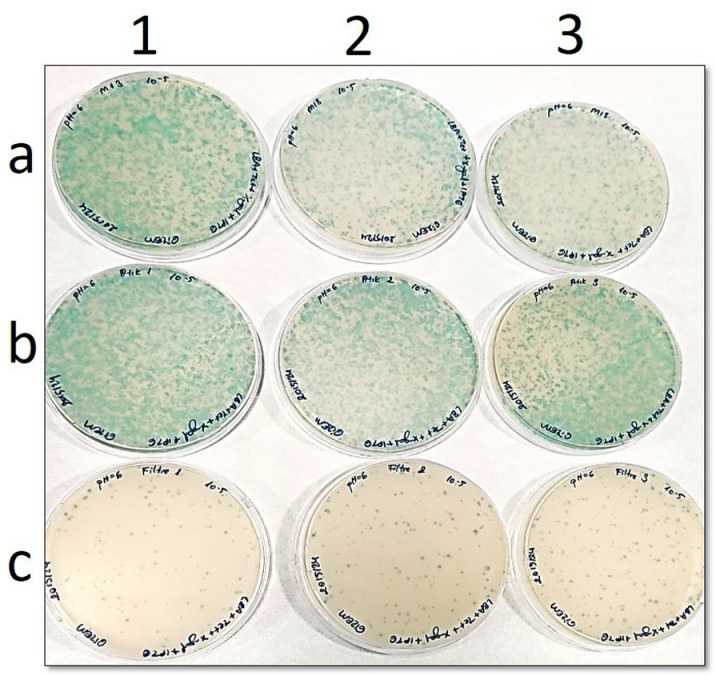
Comparison of phage numbers by the Faj-elek method at pH 6. a) Before filter treatment; b) Phages passing to the lysate; c) Phages remaining on the filter. 1,2, and 3 shows the number of repetitions.

**Fig 4:**
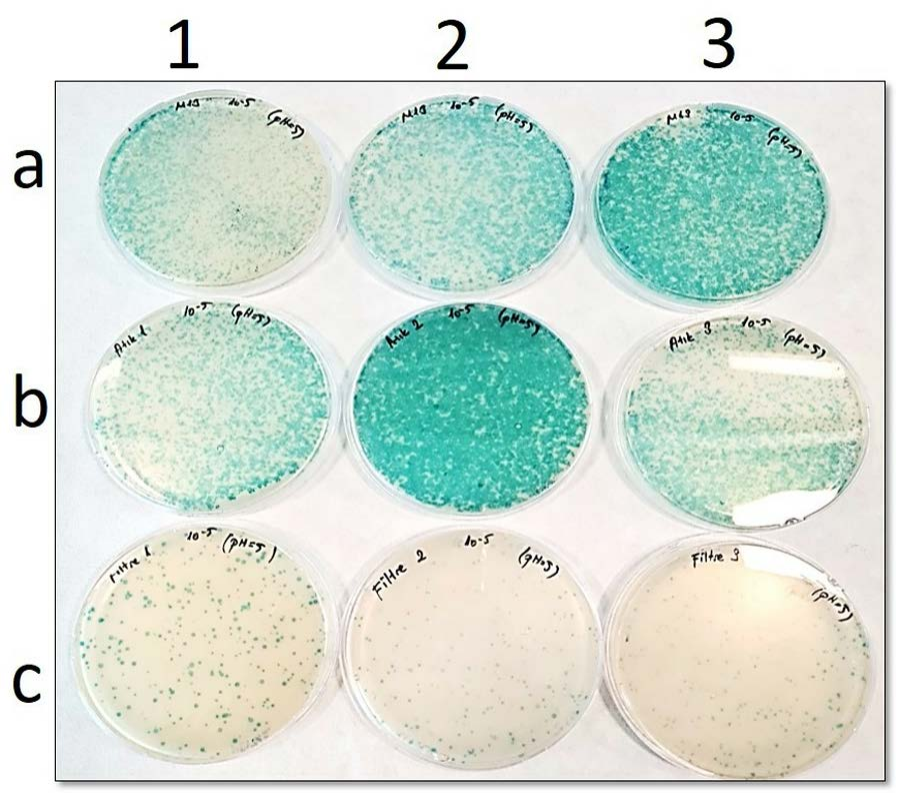
Comparison of phage numbers by the Faj-elek method at pH 5. a) Before filter treatment; b) Phages passing to the lysate; c) Phages remaining on the filter. 1,2, and 3 shows the number of repetitions.

**Fig 5:**
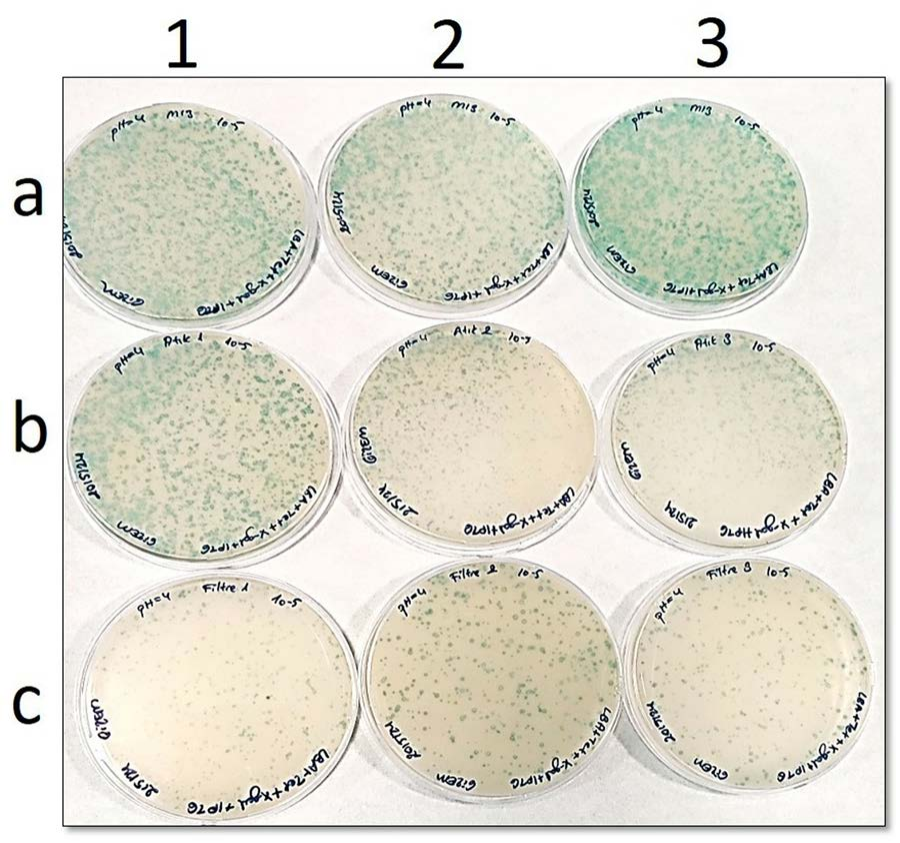
Comparison of phage numbers by the Faj-elek method at pH 4. a) Before filter treatment; b) Phages passing to the lysate; c) Phages remaining on the filter. 1,2, and 3 shows the number of repetitions.

**Fig 6:**
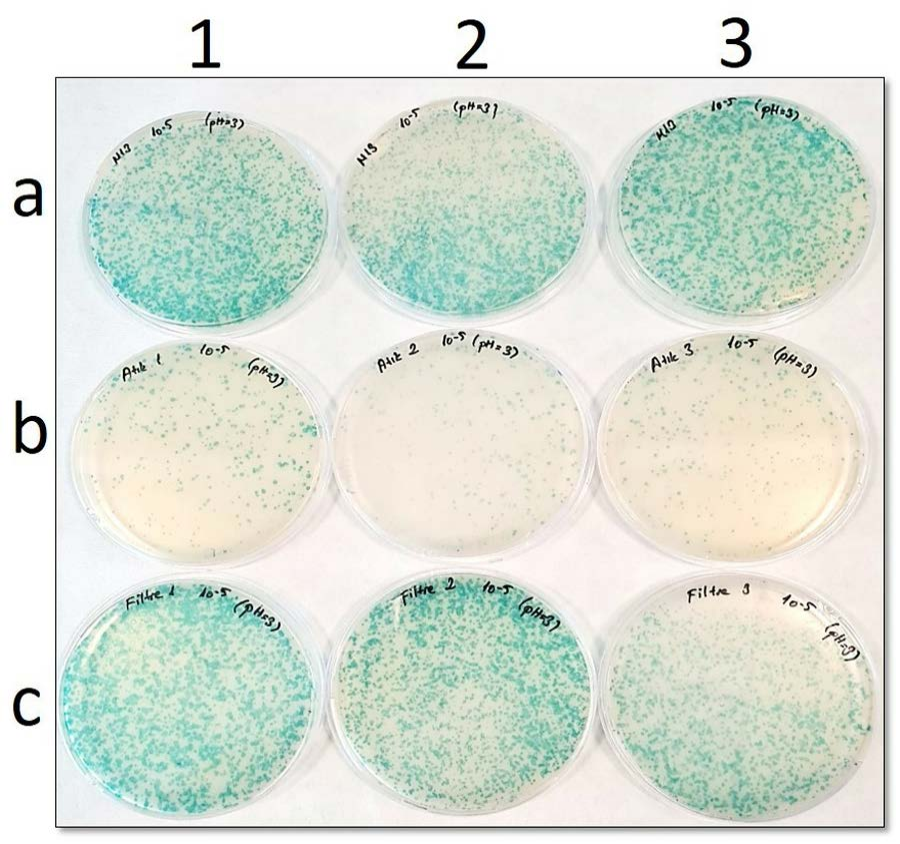
Comparison of phage numbers by the Faj-elek method at pH 3. a) Before filter treatment; b) Phages passing to the lysate; c) Phages remaining on the filter. 1,2, and 3 shows the number of repetitions.

In the petri images (Figs 2 to 6), “**a”** shows the number of phages before filtration, while “**b**” is the number of phages in the filtrate. In addition, “**c**” points out the number of phages remaining in the filter. Blue plaques represent bacterial colonies infected with phages, so a pH-dependent shift of the number of blue plaques from petri dishes “**b**” to “**c**” would indicate that the Faj-elek method worked successfully.

At pH 7, when the phage culture was passed through the filter, the number of phages remaining on the filter was 282, while in the filtrate it was 3967 (Fig 2). Based on this, a recovery rate on the filter was 7%.

At pH 6, there were 283 phages left on the filter and 3284 in the filtrate when the phage culture was passed through the filter (Fig 3). On this basis, the recovery rate on the filter was found as 8%.

At pH 5, it was found that 364 phages remained on the filter and 4203 phages were in the filtrate after passing the phage culture through the filter (Fig 4). Accordingly, the recovery rate on the filter was calculated as 8%.

At pH 4, there were 615 phages on the filter and 2077 phages in the filtrate when the phage culture was passed through the filter (Fig 5). The recovery rate on the filter was 26% on this basis.

At pH 3, it was found that 2541 phages were on the filter and 410 phages were in the filtrate after passing the phage culture through the filter (Fig 6). Based on this, the recovery rate on the filter was determined as 85%.

### Comparison of methods Faj-elek and PEG

**Fig 7:**
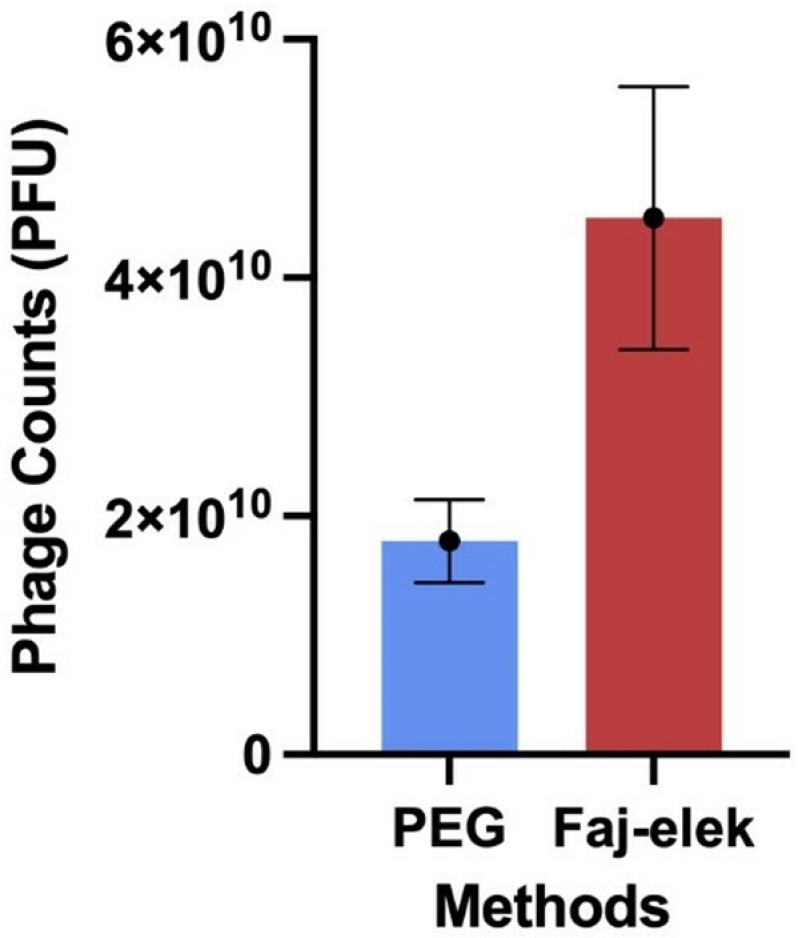
Comparison of phage counts by PEG and the Faj-elek methods.

Comparison of the total number of phages obtained using both the PEG and the Faj-elek methods for the same initial quantity was performed. Recovered total number of phages enumerated as the average of three replicates was 1.79x10^10^ PFU with the PEG method. In contrast, 4.5x10^10^ PFU were obtained as a result of the average of three replicates with the Faj-elek method (Fig 7). Based on this, it was calculated that 250% more phages were obtained by using the the Faj-elek method. In order to compare the difference between the PEG Method and the Faj-elek Method, a one-way ANOVA test was conducted. The p-value was calculated as 0.015 which indicates that the difference between the two methods is statistically significant since the p-value is less than 0.05. Therefore, there is a real and non-random difference between the two methods.

### SEM and elemental analysis

SEM was used to prove that aggregation of phages at pH 3 caused them to remain on the filter.

**Fig 8:**
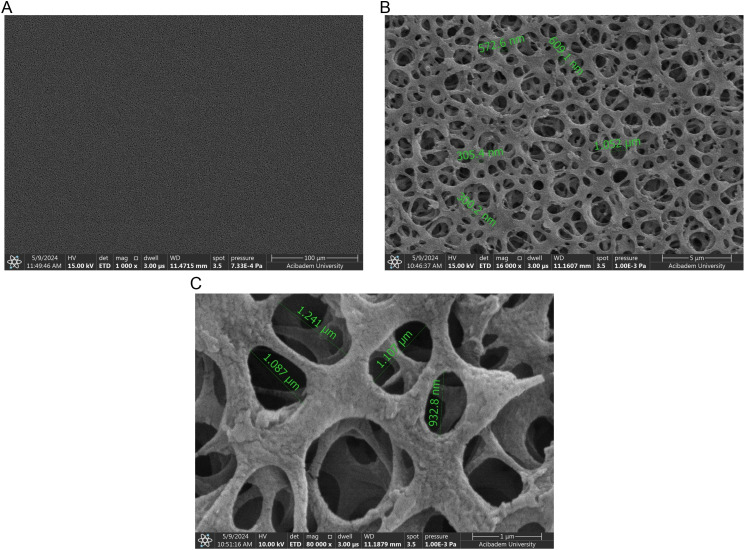
SEM images of the non-treated filter (blank). In the figure, A, B, and C represent magnifications of 100 µm, 5 µm, and 1 µm respectively.

The empty filter was used as a blank (Fig 8A–C). These results show that there are pores of very different sizes on the filter surface. Therefore, considering the size of the phages, it is expected that they can pass through these pores to the filtarate in the absence of aggregation.

**Fig 9:**
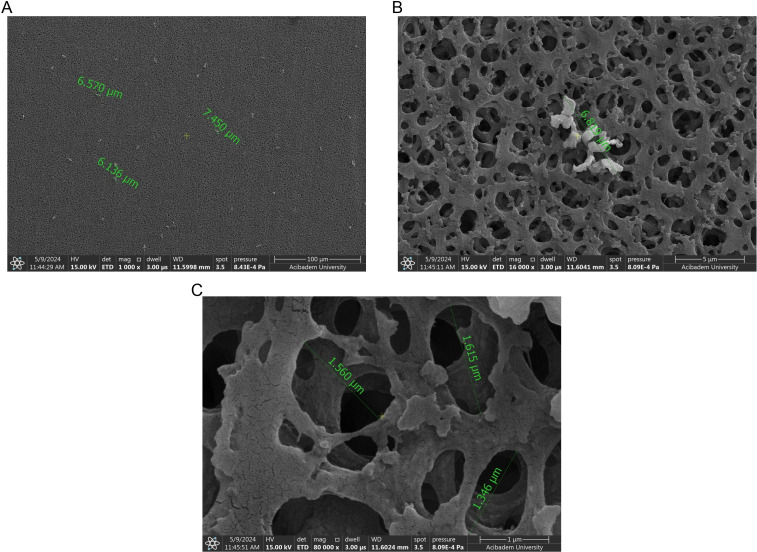
SEM images of the filter through which phages are passed at pH 7. In the figure, A, B, and C represent magnifications of 100 µm, 5 µm, and 1 µm respectively.

Phage cultures at pH 7 were also filtered and this filter was used as another control for SEM images. When the phage culture in pH 7 was filtered through the filter, salt crystals about 7 µm in size were observed (Fig 9A). At 7 µm magnification, the salt crystals were more clearly visible (Fig 9B). At higher magnification (Fig 9C), it was observed that the filter pores were open similarly to the empty filter pores in Fig 8C. There was no pore blockage due to aggregation of phages in this filter. This confirms the results of phage counting which showed phages passed through the filter.

**Fig 10:**
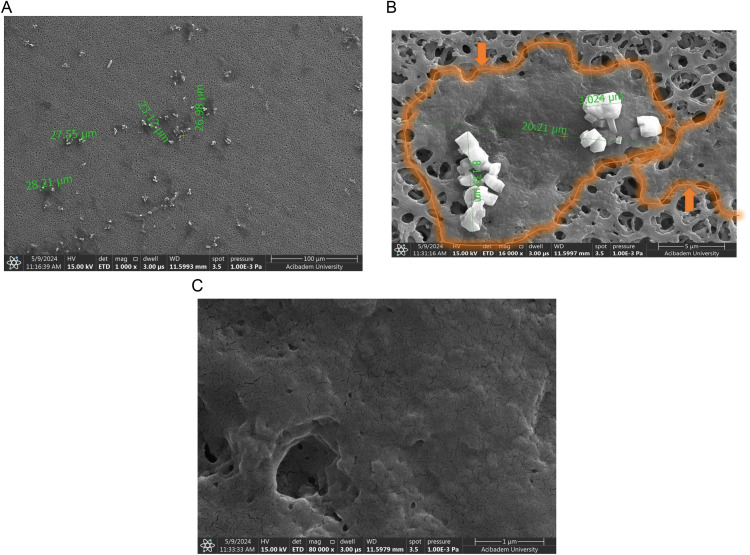
SEM images of the filter through which phages are passed at pH 3. In the figure, A, B, and C represent magnifications of 100 µm, 5 µm, and 1 µm respectively.

After the phage culture in pH 3 was filtered through the filter, aggregates of 27 µm on average were observed unlike the controls (Fig 10A). When magnified to 5 µm, these structures were more clearly visualized with salt crystals on them (Fig 10B). The size of the salt crystals in both pH 7 and pH 3 samples were approximately 3–8 µm (Figs 9B and 10B). The area surrounded by an orange line and indicated by an orange arrow shows phage aggregations (Fig 10B). Examination of this formation on further magnification revealed that it completely blocked the pores of the filter (Fig 10C). It was shown that these aggregates larger than about 20 µm could not pass through the pores of the filter and remained on the surface, which confirms the results of phage counting.

**Fig 11:**
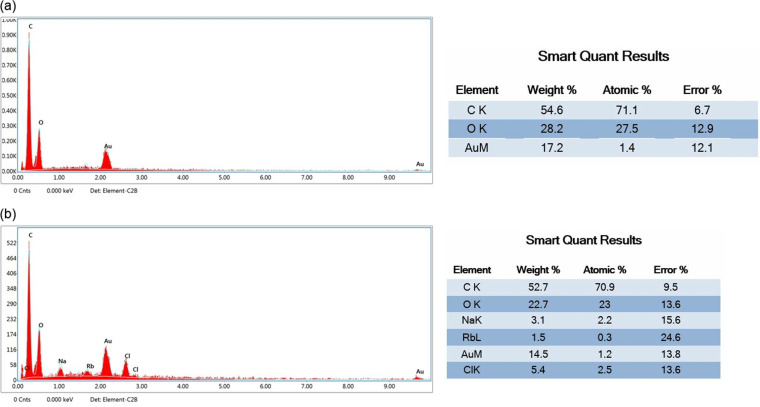
Elemental analysis. a) the non-treated filter (blank) and b) filter through which phages are passed.

In addition, an elemental analysis was also performed on the phage-passed filters and blank filters (Fig 11a and b). Accordingly, the blank filter had C and O elements were 54.6% and 28.2, respectively, while the phage-passed filter had 52.7% and 22.7% of these elements. Since the filter is made of cellulose acetate, such high C and O in both the blank filter and the treated one was as expected. Additionally, in the phage-passed filter, Cl, Na and Rb elements were detected in 5.4%, 3.1%, and 1.5% ratios, in which Na and Cl originates from the LB medium.

## Discussion

M13 bacteriophages are very important in molecular biology, biotechnology and nanotechnology [5,24–26]. In particular, phage display technology, which plays a critical role in the development of both diagnostic and therapeutic tools such as drug delivery, has been awarded a Nobel Prize [4].

In the large-scale production of M13 phages in research or industrial settings, bacteria are utilized as factories, and phages are obtained through purification from these phage cultures. Several different methods have been developed for the purification or concentration of phages [7].

Density gradient ultracentrifugation based on the use of cesium chloride is one of the methods that enables high purity phages to be obtained [7,27]. However, it is difficult to use in every laboratory as it requires a centrifugation speed of 40,000–100,000xg making it expensive in terms of equipment [7]. It is also not suitable for high volume phage cultures due to volume limitations for ultracentrifugation [7].

Other options for phage purification include size-exclusion chromatography and ion exchange chromatography [28–30]. High performance liquid chromatography (HPLC) or fast protein liquid chromatography (FPLC) systems using columns on which phages are attached are quite costly and are not available in every laboratory, so their use is limited [7].

Finally, there are methods based on precitipitation for the purification and concentration of phages. These include the use of non-ionic polymers such as polyethylene glycols (PEG) and multivalent cations such as spermidine as well as isoelectric-based precipitation [7]. Similar to the salting-out of proteins in the presence of monovalent salts such as NaCl, PEG molecules help phages to form aggregates and escape from the solution and consequently to be precipitated and purified by centrifugation [7]. PEG precipitation is one of the most widely used methods for phage purification and concentration, as it can be applied at much lower cost compared to the expensive equipment of ultracentrifugation or chromatography systems [7]. Despite these gains, there is a high contamination rate due to the use of large amounts of PEG and 2.5 M NaCl, which co-precipitate with the phage pellet [7]. In addition, bacterial enzymes such as RNAase can also be one of the co-precipitated molecules, which can cause interference in the downstream use of phages. CsCl gradient centrifuge method is usually used for further purification after the PEG method [11]. Furthermore, in the study published by Carroll-Portillo et.al. (2021), it was shown that the use of PEG reduced M13 phage activity by 7.7%−53.4% and the use of CsCl by 43%−55.1% [23]. The surface charges of filamentous phages were studied by polyelectrolyte titration method and it was determined that their isoelectric point was 4.2 [12]. In this case, the phages can be purified by centrifugation and precipitation once the pH of the phage culture is lowered to the point where their net charges are zero [13,14]. However, charged residues in biomolecules such as DNA in the culture are neutralized by the oppositely charged residues of phages and therefore they co-precipitate with phages [12–14]. As a result, purified phages still contain about 75% of the DNA molecules of the culture [13]. There are also concerns about the possibility of co-precipitation of cultured proteins with the same pI [7]. In this respect, this method has some limitations, although it is quite simple to perform.

All methods for concentrating or purifying M13 phages, which are very important in molecular biology and biotechnology, are as listed above. However, they have important limitations such as impurities or the requirement for the use of expensive and complicated devices.

In this study, we have developed a new method (the Faj-elek Method) to obtain M13 bacteriophages quickly, simply and purely using a filter to overcome all these disadvantages.

A study by Katayama et al. (2014) was performed by filtration of polioviruses in pure water and seawater samples through negatively charged membranes and elution of viruses with 1 mM NaOH (pH 10.5) after washing the filter with 0.5 mM H_2_SO_4_ (pH 3.0) [31]. As a result of the study using negatively charged membranes, the average recovery efficiency from artificial seawater was calculated as 62% and the efficiency was increased compared to the 6% recovery with positively charged membranes [31]. This result is related to the fact that phages remain on the filters as a result of their aggregation at low pHs, which was shown by Langet et.al. (2007) in studies with MS2 bacteriophages [32].

We therefore investigated the feasibility of remaining of M13 bacteriophages by aggregation on syringe filters with a 0.22 µm in pore diameter and then reverse the filter to recover the retained phages. The cellulose acetate was chosen as the filter membrane used because it has the ability to adsorb charged molecules through electrostatic interactions because it has a negative charge [33].

For proof of concept, the phage culture was first diluted 10^-5^-fold in LB medium to eliminate dilution error due to aggregation and then the media were adjusted to different pH values (between 3 and 7). The filters were conditioned with LB medium at the pHs tested and then phage cultures were passed through the filters. The filters were reversed and phages were recovered by flushing the TBS buffer through the filters in the opposite direction. Phage counts in both the initial culture before filter application, in the filtrate and in the recovery sample were determined by the top agar method. Accordingly, the recovery rate between pH 7–3 was between 7%−85%. This showed that the Faj-elek Method worked effectively.

After proof of concept, the whole phage culture was investigated to work with the the Faj-elek Method without dilution. It was observed that phages could be recovered by reversing the filter in the most of the replicates, while in some replicates, phages were not recovered either in the lysate or on the filter (S1–S3 Figs). This might be related to the findings of Langet et.al. (2007) who reported that when low pH decreases the number of PFUs, this is due to adhesion, inactivation and aggregation [32]. Therefore, these results might be due to the aggregation of phages in high phage concentrations at low pH affected their abilities to infect the bacteria and greatly reduces their numbers as PFUs.

To show that phages are retained on the filter surfaces as a result of aggregation due to lowering the pH when phage density is high, SEM images of the membranes were taken and it was shown that phage aggregations were located on the filter surface in the form of large spots an average of 27 µm.

To prevent unreleasable attachment of phages to the filter, the ionic strength of the medium was increased and for this purpose, NaCl was added to the dense phage culture with a final concentration of 1.5 M and the filter was also conditioned under the same conditions. As a result of the addition of salt, the recovery rate increased. As a result of a comparison with the standard PEG method, it was determined that the number of phages obtained with the Faj-elek Method was 250% higher.

Bacteriophages with icosohedral structure are between 100–500 nm in length while those with filamentous structure are 500–2000 nm [34]. Considering that M13 phages are 900 nm in length, it seems possible that this new method can be used for the purification of other bacteriophages, especially filamentous ones.

As a conclusion, these results show that as an alternative to existing methods, the Faj-elek Method offers a cheaper, faster and simpler way to purify M13 phages, which are widely used in molecular biology and biotechnology, and can be easily used in any laboratory.

## Supporting information

S1 Fig Comparison of phage numbers in high phage concentrations by the Faj-elek method. a) Before filter treatment; b) Phages remaining on the filter; c) Phages passing to the lysate. 1,2, and 3 shows the number of repetitions.
(TIF)

S2 Fig Comparison of phage numbers in high phage concentrations by the Faj-elek method. a) Before filter treatment; b) Phages remaining on the filter; c) Phages passing to the lysate. 1,2, and 3 shows the number of repetitions.
(TIF)

S3 Fig Comparison of phage numbers in high phage concentrations by the Faj-elek method. a) Before filter treatment; b) Phages remaining on the filter; c) Phages passing to the lysate. 1,2, and 3 shows the number of repetitions.
(TIF)

S4 Fig SEM images of the filter through which phages are passed at pH 3. It represents magnifications of 5 µm.
(TIF)
