# Supplementary figures and images for: Simple, rapid, and efficient purification of M13 phages: The Faj-elek method
Source: PLoS One. 2025 Jun 6;20(6):e0325621. doi: 10.1371/journal.pone.0325621 (PMC12143533; doi:10.1371/journal.pone.0325621)

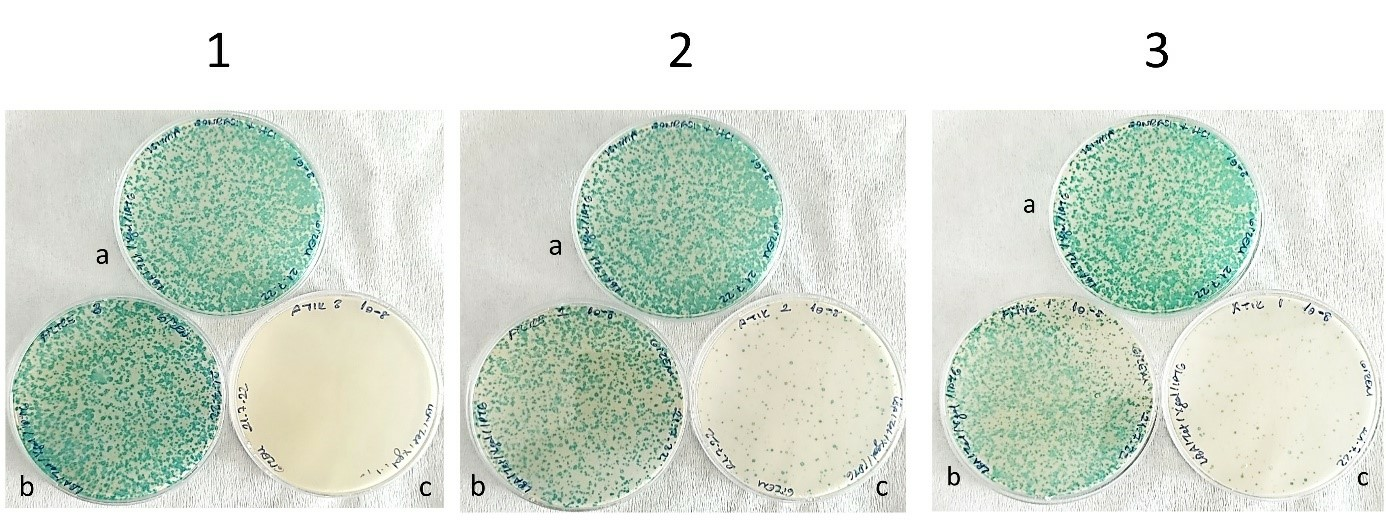

Supplement: S1 Fig — a) Before filter treatment; b) Phages remaining on the filter; c) Phages passing to the lysate. 1,2, and 3 shows the number of repetitions. (TIF) [file pone.0325621.s001.tif]

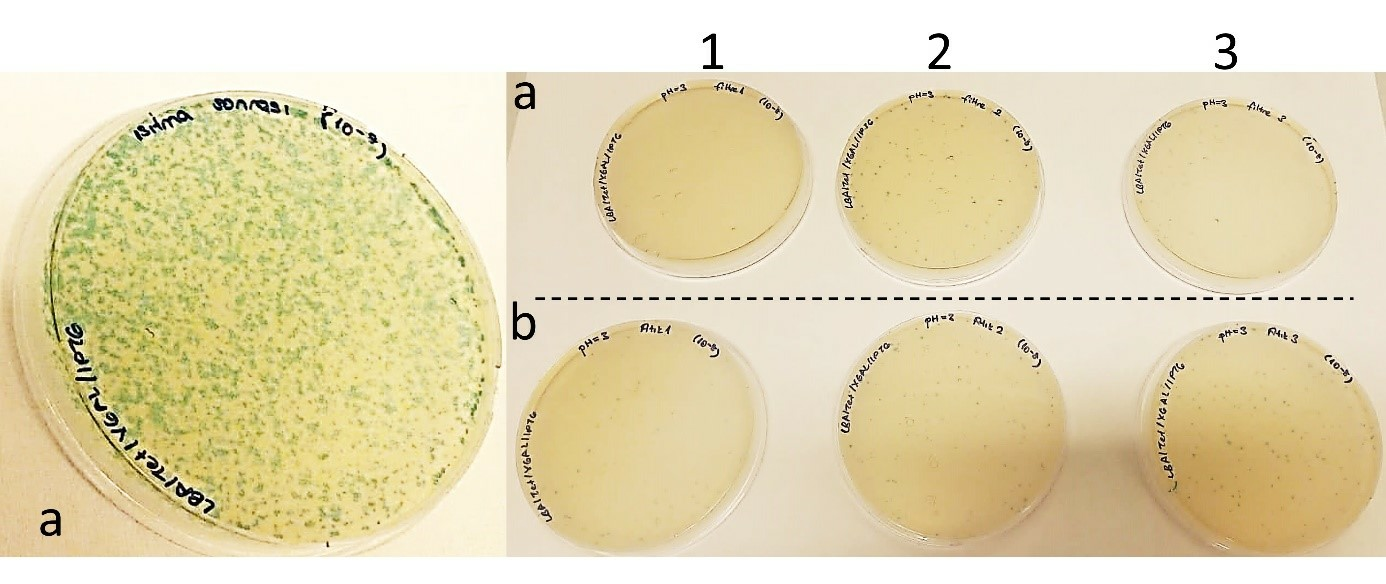

Supplement: S2 Fig — a) Before filter treatment; b) Phages remaining on the filter; c) Phages passing to the lysate. 1,2, and 3 shows the number of repetitions. (TIF) [file pone.0325621.s002.tif]

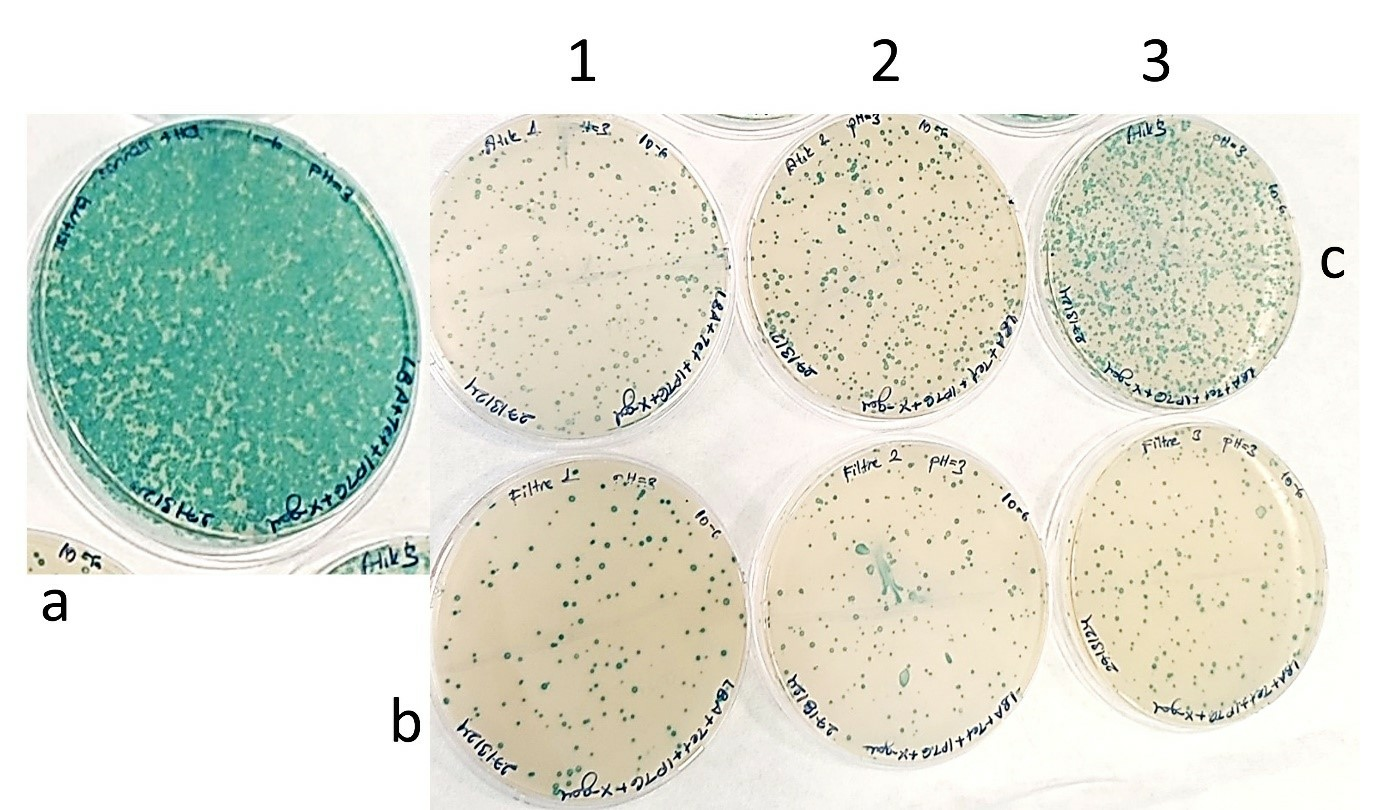

Supplement: S3 Fig — a) Before filter treatment; b) Phages remaining on the filter; c) Phages passing to the lysate. 1,2, and 3 shows the number of repetitions. (TIF) [file pone.0325621.s003.tif]

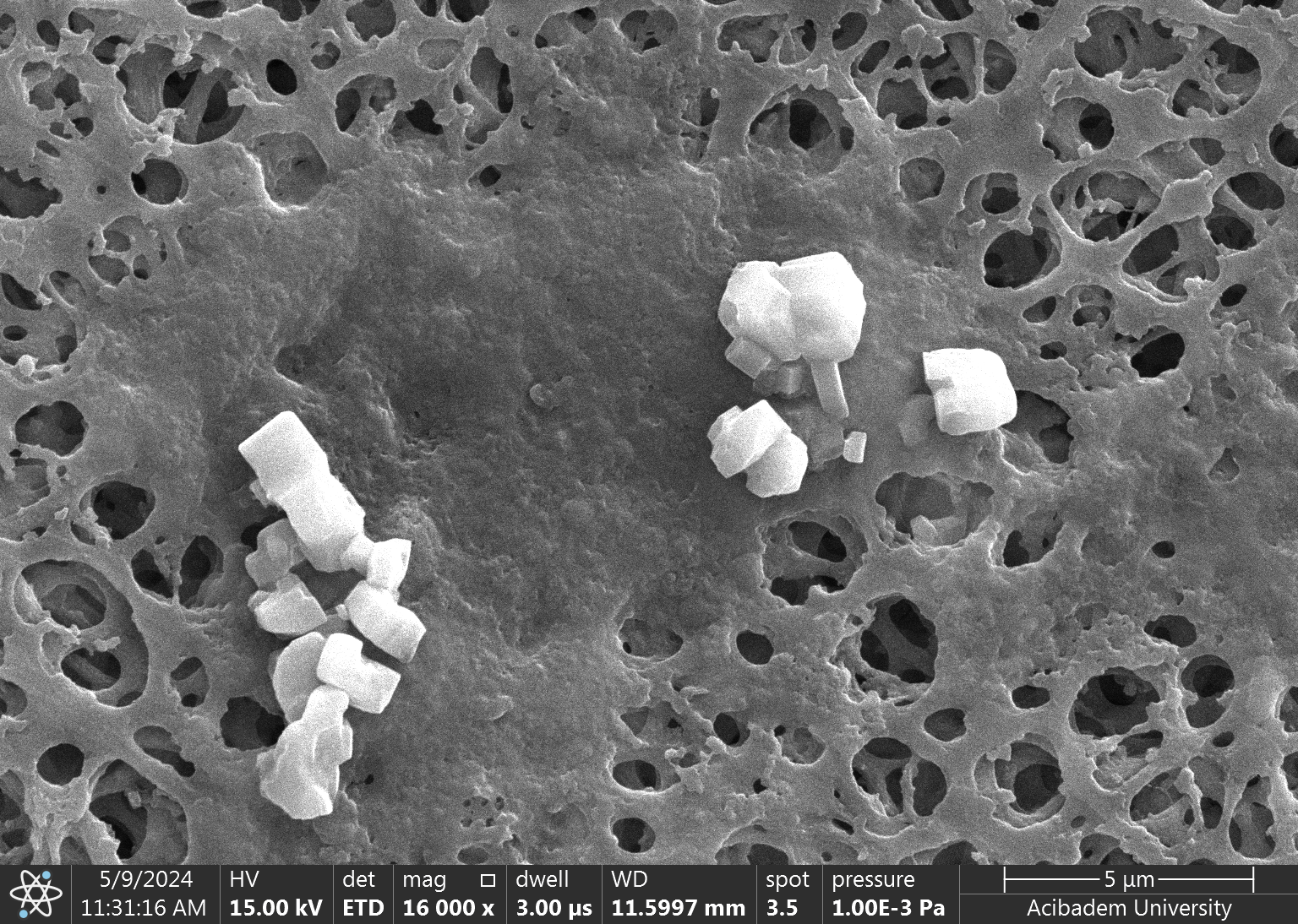

Supplement: S4 Fig — It represents magnifications of 5 µm. (TIF) [file pone.0325621.s004.tif]
